# Cryptotanshinone targets tumor-immune-microbiome axis to suppress colorectal cancer

**DOI:** 10.3389/fphar.2025.1667500

**Published:** 2025-12-09

**Authors:** Zhenya Yang, Mengting Zhou, Fenglin Luo, Shiqi Feng, Yu Tan, Qiqian Wang, Zeneng Cheng, Ruirong Tan, Rui Li

**Affiliations:** 1 Xiangya School of Pharmaceutical Sciences, Central South University, Changsha, Hunan, China; 2 Translational Chinese Medicine Key Laboratory of Sichuan, Sichuan-Chongqing Joint Key Laboratory of Innovation of New Drugs of Traditional Chinese Medicine, Sichuan Institute for Translational Chinese Medicine, Sichuan Academy of Chinese Medicine Sciences, Chengdu, China; 3 School of Pharmacy, Chengdu University of Traditional Chinese Medicine, Chengdu, China; 4 Department of Molecular and Clinical Cancer Medicine, University of Liverpool, Liverpool, United Kingdom; 5 Department of Radiation Oncology, Radiation Oncology Key Laboratory of Sichuan Province, Sichuan Clinical Research Center for Cancer, Sichuan Cancer Hospital & Institute, Sichuan Cancer Center, University of Electronic Science and Technology of China, Chengdu, China

**Keywords:** colorectal cancer therapy, natural products, tumor microenvironment, gut microbiota, immune checkpoint, metabolic reprogramming

## Abstract

**Background:**

Colorectal cancer (CRC) progression involves complex interactions between tumor cells, immune evasion, and metabolic reprogramming. Cryptotanshinone (CTS), a bioactive diterpenoid from *Salvia miltiorrhiza*, has demonstrated anticancer potential, but its integrated effects on CRC remain unclear.

**Methods:**

We employed both *in vitro* and *in vivo* models to evaluate the therapeutic effects and mechanism of CTS. Using MC38 cells and mouse-derived CRC organoids, we assessed its impact on proliferation and apoptosis through CCK-8, clonogenic, and Annexin V/PI assays. For *vivo* evaluation, a murine AOM/DSS-induced CRC model was established and administered CTS via intraperitoneal injection for 8 weeks. Comprehensive analyses included histopathology, immune profiling by flow cytometry, 16S rRNA sequencing of gut microbiota, and LC-MS-based metabolomics.

**Results:**

CTS exerted potent anti-CRC effects, suppressing tumor cell proliferation and inducing apoptosis *in vitro*. In AOM/DSS-induced mice, CTS significantly inhibited tumor growth, ameliorated colon shortening and pathological damage, and reduced inflammation. Mechanistically, CTS alleviated T cell exhaustion, reversed metabolic dysregulation, and restored gut microbiota community structure.

**Conclusion:**

CTS effectively suppresses CRC progression. Its efficacy is associated with the coordinated modulation of the tumor-immune-microbiome axis, involving direct cytotoxicity, reduced PD-1+ T cell levels, and restructuring of the gut microbial community. These results highlight CTS as a promising multi-faceted therapeutic candidate and provide a preclinical rationale for its further development.

## 1 Introduction

Colorectal cancer (CRC) is a malignant tumor of the gastrointestinal tract with the second highest mortality rate and the second highest incidence rate of malignant tumours in the world (Siegel et al., 2024). CRC is a heterogeneous disease caused by various pathogenic mechanisms, including gene mutations, somatic mutations, genetic instability, and epigenetic alterations (Roshandel et al., 2024). Currently, surgery remains the first-line treatment for CRC. However, with advancements in radiotherapy, chemotherapy, targeted therapy, and immunotherapy, a multidisciplinary treatment approach has become integral throughout the course of CRC management. Despite these developments, a significant proportion of patients, particularly those with advanced disease, eventually develop recurrence and metastasis (Shi et al., 2023; Yang et al., 2021). Given these limitations, it is imperative to explore the pathogenic mechanisms of CRC and to develop novel and effective therapies for its treatment.

Emerging evidence highlights the critical role of gut microbiota in CRC pathogenesis, as dysbiosis can weaken the intestinal barrier and promote carcinogenic processes (Stefani et al., 2021). For instance, harmful bacteria such as *Escherichia coli* and *Bacteroides fragilis* can trigger inflammation via pathways like NF-κB, leading to epithelial damage and DNA mutations (Liu et al., 2023). Gut microbiota metabolites further influence CRC development by modulating inflammation and immune responses (Privitera et al., 2021). Therefore, understanding the interplay between gut microbiota and the inflammatory microenvironment is crucial for identifying biomarkers for CRC prognosis and developing therapeutic strategies aimed at restoring microbiota balance to prevent and treat this malignancy.

Cryptotanshinone (CTS) is a lipophilic active compound extracted from the traditional Chinese medicine Danshen (*Salvia miltiorrhiza* Bunge). Increasing evidence confirms that CTS exhibits potent inhibitory effects on various types of tumor cells (Chen et al., 2013). For instance, CTS can suppress the expression of matrix metalloproteinases 2/9 (MMP2/9) and inhibit the migration and proliferation of non-small cell lung cancer through the PI3K/Akt/mTOR signaling pathway (Zhang J. et al., 2018). Reports indicate that CTS targets hypoxia-inducible factor-1α (HIF-1α) and inhibits its nuclear translocation, thereby blocking the growth and progression of CRC (Zhang L. et al., 2018). However, the precise mechanism by which CTS antagonizes CRC remains insufficiently understood, and it is still unclear whether CTS exerts its anti-CRC effects by influencing the composition and distribution of gut microbiota and their metabolites. Therefore, this study utilized an AOM/DSS-induced CRC model in Balb/c mice to evaluate the effects of CTS intervention. Following CTS administration, 16S rRNA sequencing was conducted to analyze gut microbiota, and LC-MS metabolomics was employed to examine metabolic profiles. This approach aimed to investigate whether CTS inhibits the initiation and progression of CRC, and if so, to explore its association with changes in gut microbiota, metabolites, and inflammatory factors within the intestinal microenvironment. The findings are expected to provide experimental foundations and theoretical insights for identifying new biomarkers and potential candidate compounds for CRC prevention and treatment.

## 2 Materials and methods

### 2.1 Chemicals and reagents

Cryptotanshinone (CTS), sourced from MedChemExpress, was stored at 4 °C for future use. AOM was procured from Sigma-Aldrich (Massachusetts, United States), while DSS was obtained from MP Biomedicals, LLC (Santa Ana, United States). The Ki-67 rabbit antibody was acquired from Proteintech Group, Inc. (Chicago, United States). For cytokine detection, the mouse IL-6 ELISA kit (96 tests), mouse IL-1β ELISA kit (96 tests), and mouse TNF-α ELISA kit (96 tests) were purchased from Multi Sciences (Lianke) Biotech Co., Ltd. (Hangzhou, China). Furthermore, various antibodies were obtained from BioLegend (San Diego, CA, United States), including PerCP/Cyanine5.5 anti-mouse CD3, FITC anti-mouse CD4, APC/Fire™ 750 anti-mouse CD8a, PE anti-mouse CD107a (LAMP-1), Brilliant Violet 421™ anti-mouse CD279 (PD-1), and APC anti-mouse CD28.

### 2.2 Cell and organoid culture

The MC38 CRC cell line was maintained in Dulbecco’s Modified Eagle Medium (Biosharp, Beijing, China) containing 10% fetal bovine serum and 1% penicillin/streptomycin and incubated at 37 °C with 5% CO_2_. CRC organoids were derived from the AOM/DSS-induced *in situ* CRC model, were embedded in Matrigel (Corning, NY, United States) and cultured in a 37 °C incubator with cytokine-enriched conditioned medium. The organoids were subcultured every 7 days.

### 2.3 Cell viability and colony formation assays

Cell viability was assessed using the Cell Counting Kit-8 (CCK8) (Abbkine Scientific Co., Ltd, Wuhan, China). A total of 8,000 cells were plated into each well of a 96-well plate and exposed to various drug concentrations for 24, 48, and 72 h. Absorbance at 450 nm was then measured. For colony formation assays, 800 cells were seeded into a 6-well plate, with medium changes every 3 days. Treatment was stopped once colonies reached a size of more than 50 cells. The colonies were then fixed with 4% paraformaldehyde and stained using crystal violet. Colony sizes were analyzed using ImageJ.

### 2.4 Cell apoptosis assays

Apoptosis was assessed using the Annexin V-FITC/PI Apoptosis Detection Kit (Elabscience Biotechnology Co., Ltd., Wuhan, China). Cells (5 × 10^5^ per well) were seeded in a 6-well plate and treated for 24 h with various concentrations of CTS. After treatment, both the cells and supernatant were collected, washed with PBS, and resuspended in 100 μL of diluted 1 × Annexin V Binding Buffer. The cell suspension was then treated with 2.5 μL of Annexin V-FITC Reagent and 2.5 μL of PI Reagent. The mixture was incubated for 15–20 min at room temperature, protected from light. After incubation, 400 μL of diluted 1 × Annexin V Binding Buffer was added, and the samples were analyzed by flow cytometry.

### 2.5 Organoid drug sensitivity assays

Organoids were dissociated using TrypLE Express (Gibco BRL, Grand Island, NY, United States) and passed through a 100 μm cell strainer. A total of 1,000 cells were plated in 9 μL droplets per well in a 96-well plate. After 2 days of organoid culture, the medium was replaced with one containing the drug. Following a six-day incubation, cell viability was assessed by CCK8 assays, and OD values were measured at 450 nm. Changes in organoid number and size following treatment were observed and documented microscopically.

### 2.6 Animals and treatment regimens

Seven-week-old male Balb/c mice, with an average weight of approximately 20 ± 2 g, were sourced from Chengdu Dashuo Laboratory Animal Co. (Chengdu, China). All animal experiments were approved by the Medical Ethics Committee of Chengdu Medical College and performed in accordance with institutional guidelines. The AOM/DSS model in mice was developed based on previously documented methods (Zhou et al., 2025). Following a one-week acclimatization period in a specific pathogen-free (SPF) environment, the mice received a single intraperitoneal injection of AOM at a dosage of 12.5 mg/kg body weight. One week later, they were given drinking water containing 2% DSS for a week, followed by 2 weeks of regular water. This treatment cycle, lasting 3 weeks, was repeated three times over a total of 9 weeks. The mice were then separated into three groups: the negative control group, the AOM/DSS model group, and the CTS group, which received intraperitoneal injections of CTS at 50 mg/kg/day. The control and AOM/DSS groups were administered an equivalent volume of saline as a placebo. Throughout the study, body weight was monitored every other day, and fecal samples were assessed for blood. At the conclusion of the experiment, the mice were provided with regular water and fasted for 24 h before sampling. Body weight and the weights of the liver, thymus, and spleen were recorded. Additionally, colorectal length was measured, and blood, feces, colonic contents, and colorectal tissue were collected for further analysis.

### 2.7 Hematoxylin and eosin (H&E) staining of CRC tissues of mice

Colorectal tissue samples were preserved in 4% paraformaldehyde and subsequently placed in embedding cassettes. The samples were rinsed overnight with running water. Following this, they underwent dehydration and clearing through a series of graded ethanol and xylene solutions. The tissues were then transferred to a wax cylinder and immersed in wax for two hours. An embedding machine facilitated the embedding procedure, resulting in paraffin-embedded tissues that were sectioned to a thickness of 3–5 µm. These sections were baked in an oven at 60 °C for 1 h. A gradient system involving xylene, ethanol, and water was used for deparaffinization and rehydration. After staining with hematoxylin and eosin, the sections were rinsed to remove excess dye, and then mounted with neutral gum. Finally, images were captured using a BX63 inverted microscope.

### 2.8 Immunofluorescence analysis

Tissue sections, sliced to a thickness of 3–5 μm, were deparaffinized and subjected to antigen retrieval. After rinsing with phosphate-buffered saline (PBS), the sections were blocked using 5% bovine serum albumin (BSA) and then incubated overnight at 4 °C with a primary anti-KI67 antibody diluted 1:125. The following day, after another wash with PBS, the sections were treated with a fluorescent secondary antibody at a dilution of 1:500 for 1 hour at room temperature, ensuring protection from light exposure. Nuclei were subsequently counterstained with DAPI. After a final rinse, the slides were mounted with an anti-fade reagent. Staining was visualized and captured at 400x magnification using a BX63 microscope (Olympus, Tokyo, Japan).

### 2.9 Enzyme-linked immunosorbent assay (ELISA) for TNF-α, IL-1β, and IL-6 in mouse serum

Cytokine concentrations in mouse serum samples were quantified using ELISA, following the manufacturer’s instructions and established methodologies from prior studies. Blood samples were drawn and centrifuged at 4 °C for 10 min at 1900 rpm to separate the serum. Using an ELISA kit, the levels of TNF-α, IL-6, and IL-1β in the serum were determined. A 100 μL aliquot of each serum sample was added to a 96-well plate pre-coated with the specific capture antibody, followed by incubation at room temperature for 1.5 h. Afterward, 100 μL of Streptavidin-HRP conjugate was added to each well, and the plate was incubated for 30 min at room temperature. Subsequently, 100 μL of substrate solution was introduced, and the enzymatic reaction was halted by adding 100 μL of stop solution. Optical density was measured at 450 nm using a microplate reader.

### 2.10 Flow cytometry

Blood samples were collected into anticoagulant tubes to prevent clotting. For each sample, 100 µL of anticoagulated whole blood was aliquoted into separate tubes, and an optimal concentration of fluorescently labeled primary antibodies (CD3^+^, CD4^+^, CD8^+^, CD28, CD107a, and CD279) was added. The tubes were incubated at room temperature for 20 min in darkness. Following incubation, 2 mL of 1× erythrocyte lysis buffer was introduced to each tube to remove red blood cells, and the mixture was left to stand at room temperature for an additional 10 min. After centrifugation at 350 *g*, the supernatant was discarded. The cells were then rinsed with 2 mL of PBS, centrifuged again under the same conditions, and the supernatant was removed once more. Finally, the cell pellets were resuspended in 500 µL of PBS, and flow cytometry was used to detect and analyze fluorescence signals.

### 2.11 16s rRNA sequencing analysis of mouse fecal microorganisms

Fecal samples from each mouse group were collected using sterile forceps and placed in 2 mL sterile cryotubes, then stored at −80 °C. Genomic DNA was isolated from the samples using the OMEGA Soil DNA Kit (D5625-01) (Omega Bio-Tek, Norcross, GA, United States), and the DNA purity and concentration were evaluated. The V3-V4 hypervariable regions of the 16S rRNA gene were amplified by PCR using primers 338F and 806R with a high-fidelity DNA polymerase. PCR products were separated on a 2% agarose gel, and target DNA fragments were retrieved using the Quant-iT PicoGreen dsDNA Assay Kit. The final products were quantified on a BioTek FLx800 Microplate Reader. For sequencing library preparation, the TruSeq Nano DNA LT Library Prep Kit (Illumina) was used, and library quality was assessed with the Agilent Bioanalyzer 2100 and the Promega QuantiFluor systems. After quality checks, sequencing was conducted. Primer sequences were trimmed using Cutadapt software, and sequence quality was further processed in QIIME 2, involving filtering, noise reduction, assembly, and removal of chimeras. The resulting amplicon sequence variants (ASVs) were identified by comparison to the Silva database (version 138) for colony analysis. The raw 16S rRNA sequencing data generated in this study have been submitted to the NCBI Sequence Read Archive (SRA) under Project Number PRJNA1366731. Corresponding BioSample and SRA metadata files have been provided to ensure reproducibility.

### 2.12 Untargeted metabolomics analysis of intestinal metabolites

Tissue samples were homogenized in 200 μL of water along with ceramic beads, followed by metabolite extraction using 800 μL of a methanol/acetonitrile solution (1:1, v/v). The homogenate was then centrifuged at 140,00× g for 20 min at 4 °C, after which the supernatant was dried in a vacuum centrifuge. The dried extracts were reconstituted in 100 μL of acetonitrile/water (1:1, v/v), centrifuged again, and the supernatant was collected for LC-MS analysis.

The metabolite analysis was conducted on an Agilent 1290 Infinity UHPLC system coupled with an AB Sciex TripleTOF 6600 quadrupole time-of-flight mass spectrometer. A 2.1 mm × 100 mm ACQUITY UPLC BEH Amide column (1.7 μm, Waters, Ireland) was used for hydrophilic interaction liquid chromatography (HILIC) separation. The mobile phase consisted of 25 mM ammonium acetate with ammonium hydroxide in water (phase A) and acetonitrile (phase B), employing a gradient elution from 95% B to 40% B within a 9-min runtime. System stability was monitored using quality control (QC) samples. Data were acquired in both positive and negative electrospray ionization (ESI) modes.

Raw data files were converted to mzXML format using ProteoWizard software, and peak alignment and extraction were processed with XCMS. Significant metabolites, identified by VIP scores above 1 and p-values less than 0.05, were annotated with reference to the Human Metabolome Database (HMDB), and pathway analysis was conducted using MetaboAnalyst.

### 2.13 Statistical analysis

Statistical analyses were conducted using GraphPad Prism 8.0 and SPSS 21.0 software. Data are reported as mean ± standard deviation (SD). The applicability of parametric tests (unpaired t-test and one-way ANOVA) was assessed considering their robustness to deviations from normality. Given the small sample size (n = 6 per group), which limits the power of formal normality testing, non-parametric tests (Kruskal–Wallis test with Dunn’s *post hoc* analysis) were also applied to verify all key significant findings. The conclusions were consistent between both approaches.

## 3 Results

### 3.1 CTS treatment inhibited CRC cell proliferation, migration, and organoid formation

**FIGURE 1:**
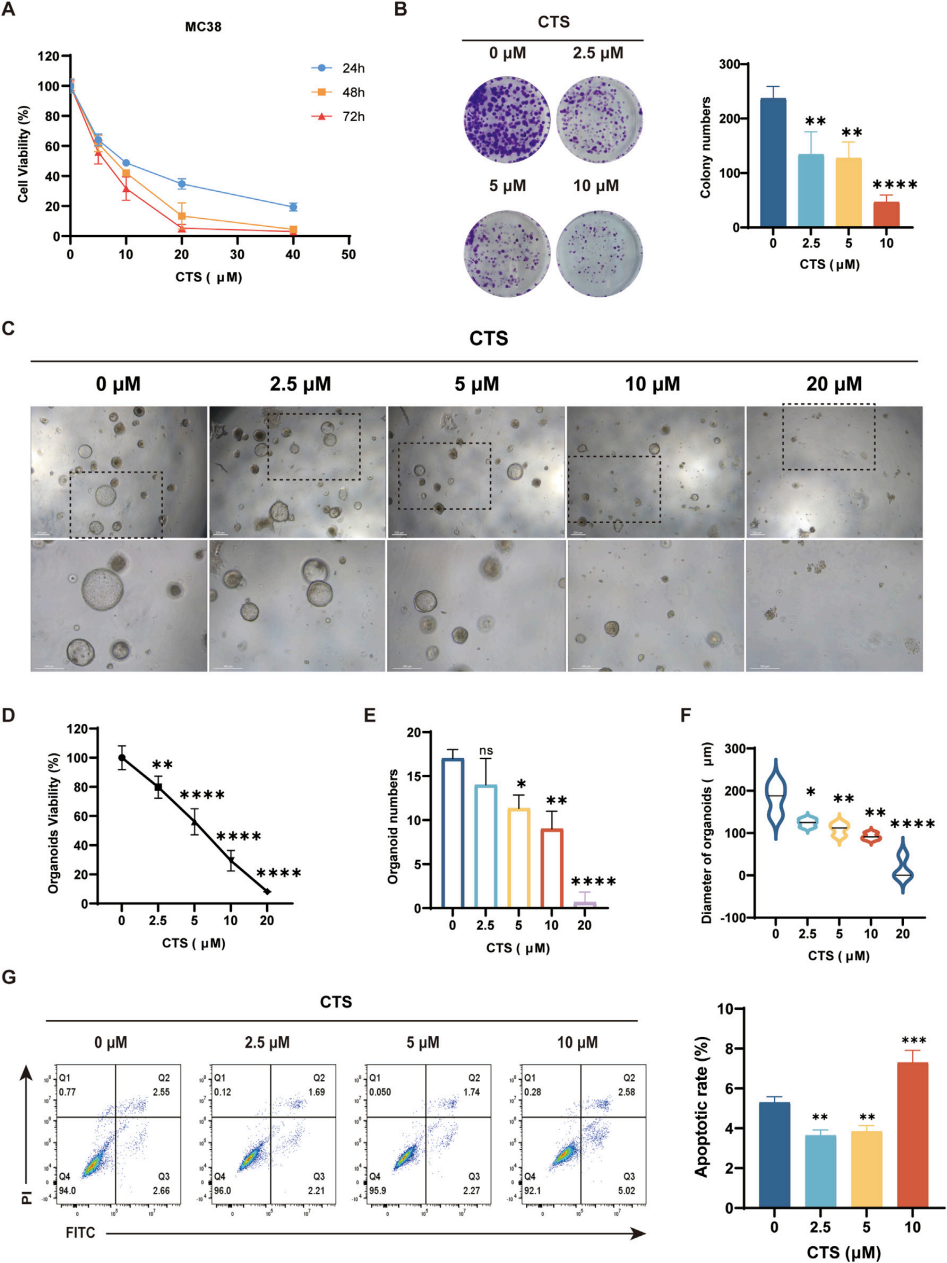
Effects of CTS treatment on MC38 cell proliferation, apoptosis, and organoid formation. **(A)** Cell viability of MC38 cells treated with CTS (0–40 µM) for 24, 48, and 72 h. **(B)** Colony formation of MC38 cells treated with CTS (0, 2.5, 5, and 10 µM). **(C)** Viability of MC38-derived CRC organoids treated with CTS (0, 2.5, 5, 10, 20 µM). **(D)** Number of MC38-derived CRC organoids after CTS treatment. **(E)** Diameter of MC38-derived CRC organoids after CTS treatment. **(F)** Representative images of MC38-derived CRC organoids treated with CTS. **(G)** Flow cytometry analysis of apoptosis in MC38 cells treated with CTS (0, 2.5, 5, 10 µM). *P < 0.05, **P < 0.01, ***P < 0.001, ****P < 0.0001.

Cell viability assays (Figure 1A) showed a significant reduction in MC38 cell viability after CTS treatment, particularly at 10 µM and above, with the strongest inhibition observed at 72 h. The colony formation assay (Figure 1B) confirmed the anti-proliferative effect of CTS, with a significant decrease in the number of colonies formed at concentrations of 5 μM and 10 µM.

Similarly, CTS treatment significantly inhibited the growth and viability of mouse-derived CRC organoids. As shown in Figures 1C–F, CTS reduced organoid viability and size in a dose-dependent manner, with the most pronounced effects observed at 10 μM and 20 µM. The number of viable organoids was significantly reduced at higher CTS concentrations. Representative images of organoids further confirmed the reduction in both organoid size and number with increasing CTS concentrations. Flow cytometry analysis (Figure 1G) showed that CTS treatment induced apoptosis in MC38 cells in a dose-dependent manner, with the highest apoptotic rate at 10 µM.

These results collectively demonstrate that CTS effectively inhibits the proliferation and migration of MC38 cells and organoids, and induces apoptosis, in a dose-dependent manner.

### 3.2 CTS treatment mitigates CRC progression and preserves colonic integrity in AOM/DSS-induced mice

**FIGURE 2:**
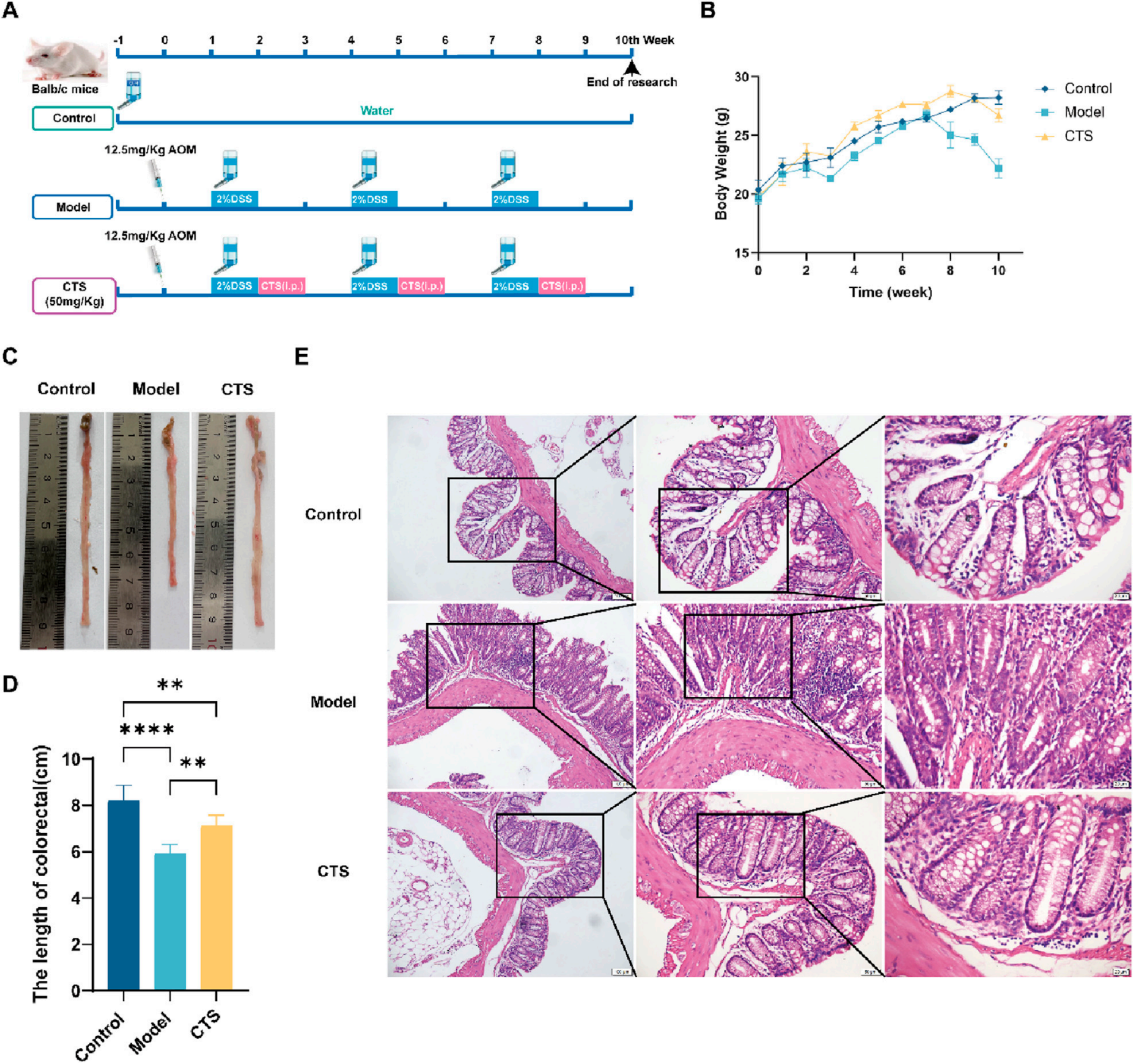
Effects of CTS treatment on CRC progression in AOM/DSS-induced Balb/c mice. **(A)** Experimental design for CTS treatment in the AOM/DSS-induced CRC mouse model. **(B)** Changes in mice’s body weight. **(C)** Comparison of tumor samples from colon tissues in macroscopic examination (n = 6). **(D)** Colorectal tissue length in mice (n = 6). **(E)** H&E staining for pathologic diagnosis. *P < 0.05, **P < 0.01, ***P < 0.0005, ****P < 0.0001.

The results demonstrate that CTS treatment effectively alleviated multiple pathological features associated with CRC progression. The 10-week experimental design (Figure 2A) shows that the CTS group received daily CTS (50 mg/kg) treatment alongside AOM/DSS to evaluate its therapeutic effects on CRC progression. As indicated by body weight trends (Figure 2B), the model group experienced significant weight loss (p < 0.05), blood in the stool, diluted stools, lethargy, and dull fur after DSS treatment. In contrast, the CTS-treated group maintained body weight comparable to the control group, suggesting that CTS ameliorated systemic manifestations of CRC.

Colonic tissue length from the cecum to the anus was also examined. As shown in Figures 2C,D, the colorectal tissue in the control group appeared smooth, flat, and resilient. The model group, however, exhibited substantial thickening, shortening (p < 0.001), reduced resilience, raised surface, and tumor-like growths. Remarkably, CTS treatment preserved colon length and restored a smoother and flatter tissue surface, suggesting a protective effect against CRC-induced structural damage.

Histopathological analysis with H&E staining provided further insights (Figure 2E). The control group displayed a well-organized intestinal lumen with regularly distributed goblet cells. Conversely, the model group showed a disorganized structure, scattered and irregular goblet cells, extensive inflammatory cell infiltration, and prominent tumor-like formations. CTS treatment significantly restored intestinal architecture, leading to a more regular intestinal lumen, reduced inflammatory cell infiltration, and smaller tumor-like structures. These results collectively suggest that CTS effectively alleviates CRC symptoms in mice by preserving intestinal structure, reducing inflammation, and mitigating tumor progression.

### 3.3 CTS treatment reduces cancer cell proliferation in AOM/DSS-induced mice

**FIGURE 3:**
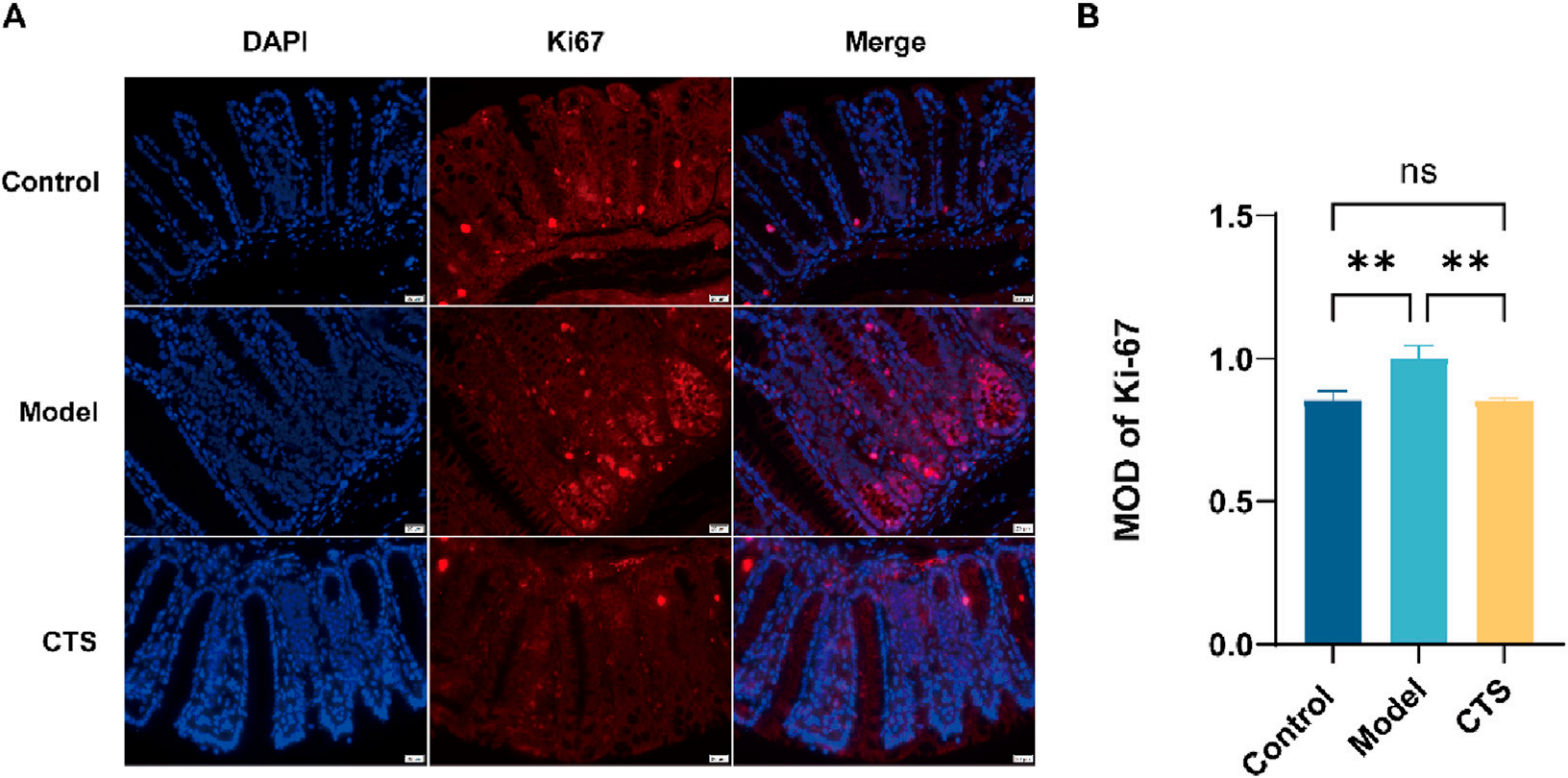
Effects of CTS treatment on cell proliferation in colorectal tissue of AOM/DSS-induced mice. **(A)** Immunofluorescence (IF) images showing Ki-67 expression in mouse colonic tissue. **(B)** Quantification of Ki-67 fluorescence intensity in colonic tissue. **P < 0.01.

To examine the effects of CTS on CRC development, Ki-67 expression was analyzed through immunofluorescence staining, as Ki-67 serves as a reliable marker for cell proliferation in both normal and cancerous tissues. The AOM/DSS model displayed a marked increase in Ki-67 levels compared to the control group, reflecting heightened cellular proliferation associated with CRC progression (Figures 3A,B). CTS treatment led to a significant reduction in Ki-67 expression (p < 0.01), indicating its role in suppressing tumor cell proliferation within this CRC model (Figures 3A,B). These results suggest that CTS may help prevent CRC progression by inhibiting excessive cell growth and proliferation.

### 3.4 CTS modulates inflammatory cytokine levels and PD-1+ T Cell population in AOM/DSS-induced CRC mice

**FIGURE 4:**
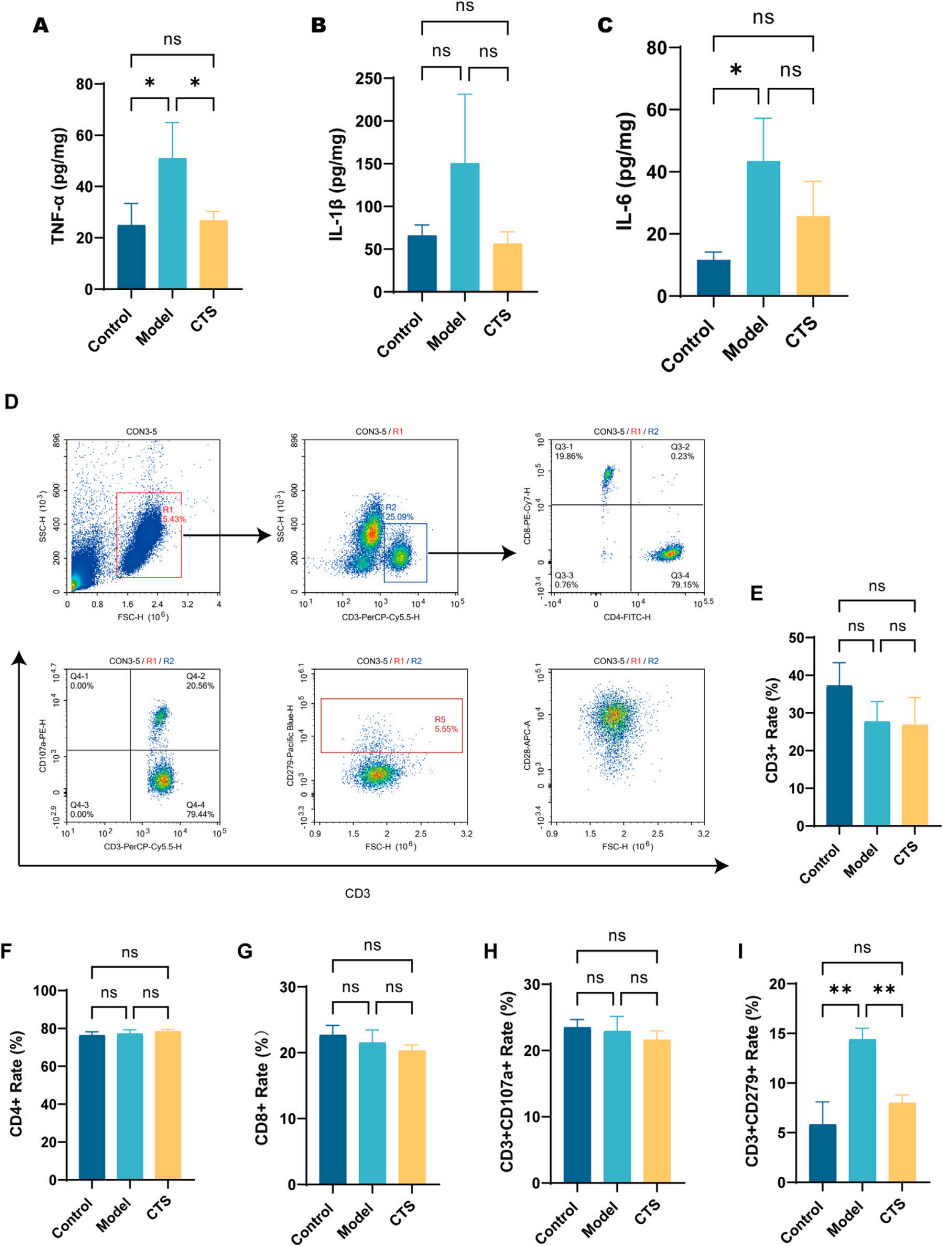
Effect of CTS on serum inflammatory cytokines and immune cell populations in AOM/DSS-induced CRC mice (n = 6). **(A)** Levels of the inflammatory cytokine TNF-α in colon tissues. **(B)** Levels of the inflammatory cytokine IL-1β in colon tissues. **(C)** Levels of the inflammatory cytokine IL-6 in colon tissues. **(D)** Flow cytometric analysis of T cell subpopulations, including CD4^+^, CD8^+^, CD3^+^CD279+ (PD-1+), and CD3^+^CD107a+ cells. **(E)** Proportion of CD3^+^ T cells. **(F)** Proportion of CD4^+^ T cells. **(G)** Proportion of CD8^+^ T cells. **(H)** Proportion of CD3^+^CD107a+ T cells. **(I)** Proportion of CD3^+^CD279+ (PD-1+) T cells. *P < 0.05, **P < 0.01.

To evaluate the effects of CTS on inflammatory cytokines and immune cell populations in AOM/DSS-induced CRC mice, we measured the levels of TNF-α, IL-1β, and IL-6, along with key immune cell subsets. As shown in Figure 4A, the levels of TNF-α were significantly increased in the Model group compared to the Control group (P < 0.05). CTS treatment significantly reduced TNF-α levels compared to the Model group (P < 0.05), restoring them to levels similar to those in the Control group. For Figure 4B, IL-1β levels showed no significant differences among the three groups (P > 0.05), though the CTS group exhibited a decreasing trend compared to the Model group. In Figure 4C, IL-6 levels were significantly elevated in the Model group compared to the Control group (P < 0.05), and CTS treatment showed a downward trend in IL-6 levels, though without statistical significance (P > 0.05).

Flow cytometry analysis provided additional insights into the immune-modulatory effects of CTS. As shown in Figure 4D, gating strategies were used to identify live cells, CD3^+^ T cells, and their subpopulations. The percentages of CD3^+^ T cells (Figure 4E), CD4^+^ T cells (Figure 4F), and CD8^+^ T cells (Figure 4G) showed no significant differences among the Control, Model, and CTS groups (P > 0.05). Similarly, the proportion of CD3^+^CD107a+ T cells, indicative of cytotoxic activity, was comparable across all groups (Figure 4H, P > 0.05).

Notably, the percentage of CD3^+^CD279+ (PD-1+) T cells, a marker associated with T cell exhaustion, was significantly elevated in the Model group compared to the Control group (P < 0.01), as shown in Figure 4I. CTS treatment significantly reduced the proportion of PD-1+ T cells compared to the Model group (P < 0.01), suggesting that CTS may alleviate T cell exhaustion in the tumor microenvironment.

In summary, while CTS treatment did not induce significant changes in the overall proportions of CD3^+^, CD4^+^, CD8^+^, or cytotoxic T cells, it showed a clear trend of reducing inflammatory cytokines and significantly alleviated T cell exhaustion, as evidenced by the reduction in PD-1+ T cells. These results suggest a potential immunoregulatory role of CTS in the AOM/DSS-induced CRC model.

### 3.5 CTS modulates gut microbial community composition in AOM/DSS-induced CRC mice

**FIGURE 5:**
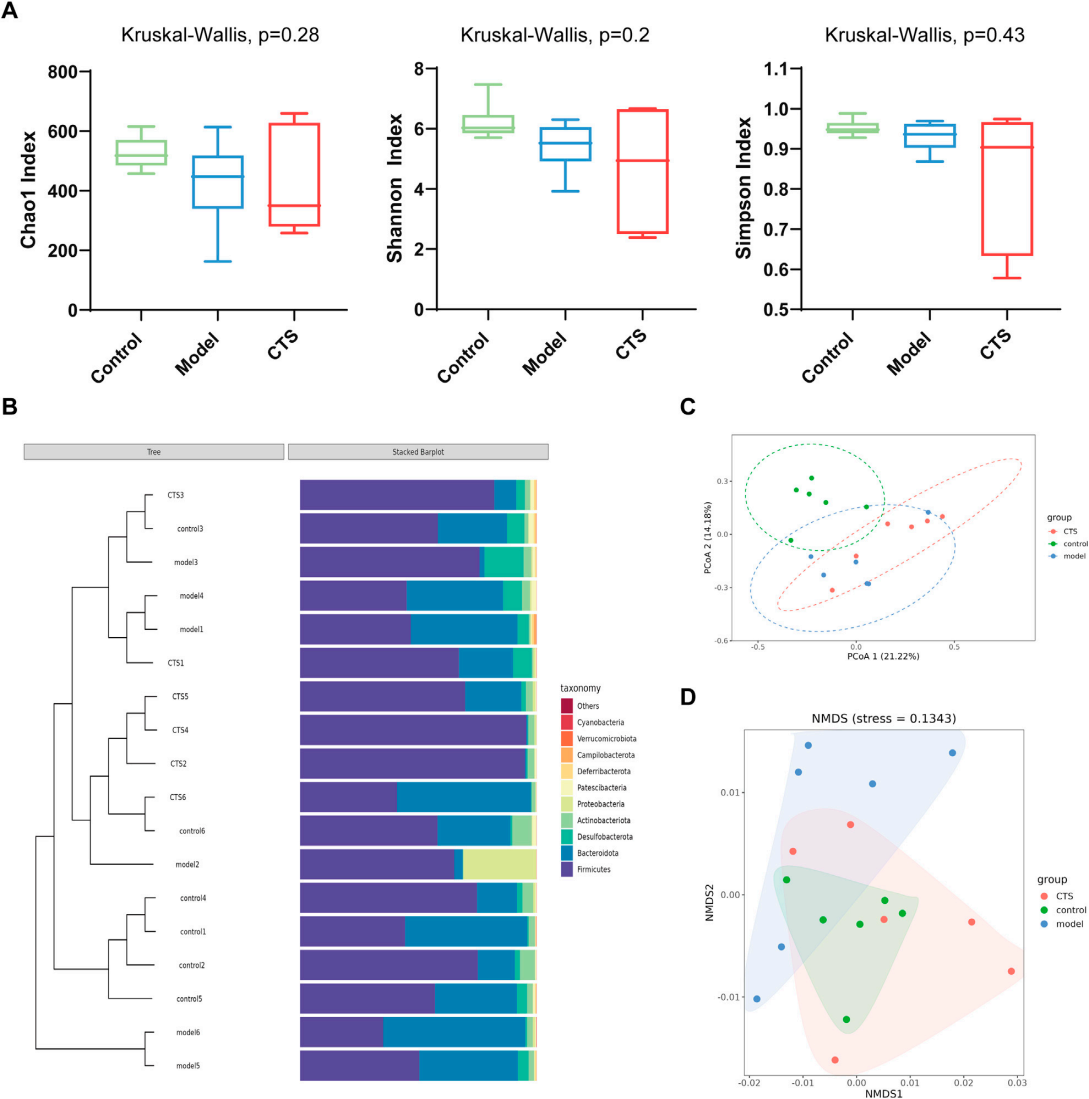
CTS Regulates Gut Microbial Community Structure in AOM/DSS-Induced CRC Mice (n = 6). **(A)** Alpha diversity indices (Chao1, Shannon, and Simpson) in fecal microbiota of Control, Model, and CTS groups. **(B)** Taxonomic composition and clustering of microbial communities at the phylum level. **(C)** Principal coordinate analysis (PCoA) of microbial composition based on Bray-Curtis distances. **(D)** Non-metric multidimensional scaling (NMDS) analysis of microbial beta diversity.

To evaluate the impact of CTS on gut microbial diversity, alpha diversity indices were analyzed. As shown in Figure 5A, the Chao1 index, Shannon, and Simpson index did not exhibit statistically significant differences among the Control, Model, and CTS groups (P > 0.05). This indicates that neither the disease model nor the CTS treatment significantly affected microbial richness or diversity at this level. Hierarchical clustering and stacked bar plots (Figure 5B) revealed distinct microbial compositions among the groups. The Control group displayed a stable cluster dominated by *Firmicutes* and *Bacteroidota*, while the Model group exhibited dysbiosis with increased Proteobacteria and reduced *Firmicutes*. The CTS group showed partial improvement, with some samples clustering closer to the Control group and a reduction in Proteobacteria compared to the Model group. Although the CTS group did not fully restore the microbial community to Control levels, its composition was more similar to the Control group than the Model group, indicating a mitigating effect of CTS on gut microbial dysbiosis. Beta diversity analyses revealed clear differences in microbial community composition among the groups. Principal coordinate analysis (PCoA; Figure 5C) demonstrated distinct clustering of microbial communities across the Control, Model, and CTS groups, with the CTS group showing a partial shift toward the Control group. Similarly, non-metric multidimensional scaling (NMDS; Figure 5D) indicated distinct clustering patterns, further suggesting that CTS modulates the overall gut microbial community structure.

**FIGURE 6:**
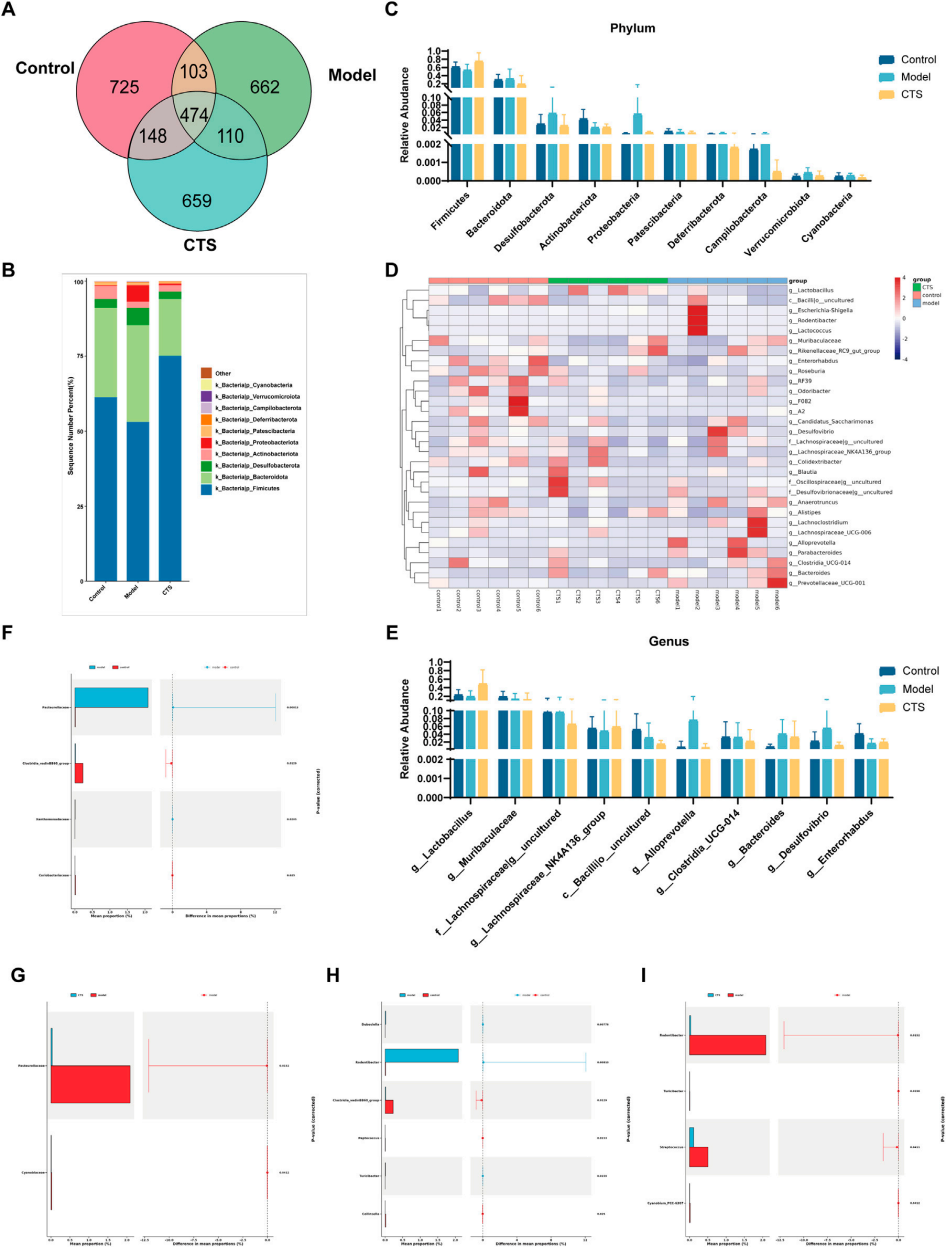
Gut microbiota composition and diversity analysis in CRC mice (n = 6). **(A)** Venn diagram illustrating the shared and unique OTUs among the three groups. **(B)** Stacked bar chart showing the relative abundance of bacterial phyla in each group. **(C)** Comparison of phylum-level relative abundances across groups. **(D)** Heatmap representing the relative abundances of genera, clustered by group. **(E)** Bar chart depicting the relative abundances of specific genera across groups. **(F,G)** Differential abundance analysis of bacterial families between Control vs. Model **(F)** and CTS vs. Model **(G)**. **(H,I)** Differential abundance analysis of bacterial genera between Control vs. Model **(H)** and CTS vs. Model **(I)**.

The Venn diagram in Figure 6A shows the shared and unique operational taxonomic units (OTUs) among the Control, Model, and CTS groups. A total of 474 OTUs were shared across all three groups, representing the core microbiota. The Control group contained 725 unique OTUs, while the Model and CTS groups exhibited 662 and 659 unique OTUs, respectively.

The dominant phyla across all groups were *Firmicutes* and *Bacteroidota* (Figures 6B,C). In the Control group, *Firmicutes* had the highest relative abundance, followed by *Bacteroidota*, *Actinobacteriota*, and *Verrucomicrobiota*. In the Model group, the relative abundance of *Firmicutes* decreased, while *Bacteroidota* increased. *Actinobacteriota* and *Verrucomicrobiota* were also reduced compared to the Control group. In the CTS group, *Firmicutes* increased and *Bacteroidota* decreased relative to the Model group, trending toward levels observed in the Control group. Additionally, *Actinobacteriota* and *Verrucomicrobiota* showed partial recovery in the CTS group.

At the genus level (Figures 6D,E), the Control group was enriched in *Lactobacillus* and *Muribaculaceae*, while the Model group showed increased levels of *Escherichia-Shigella* and *Enterobacteriaceae*, both associated with dysbiosis. In the CTS group, *Lactobacillus* and *Muribaculaceae* increased compared to the Model group but did not fully return to Control levels. Meanwhile, *Escherichia-Shigella* decreased in the CTS group relative to the Model group. Other genera, such as members of *Lachnospiraceae*, also exhibited higher abundances in the CTS group.

Heatmap analysis (Figure 6D) revealed distinct clustering patterns among the groups. The Control group clustered separately, characterized by higher levels of *Lactobacillus* and *Muribaculaceae*. The Model group formed a distinct cluster with elevated levels of *Escherichia-Shigella* and *Enterobacteriaceae*. The CTS group displayed an intermediate clustering pattern between the Control and Model groups. Genera such as *Lactobacillus* and *Muribaculaceae* in the CTS group showed trends toward Control levels, while other taxa remained closer to the Model group.

At the family level (Figure 6F), *Pasteurellaceae* was significantly enriched in the Model group compared to the Control group (p < 0.0011), while *Clostridia vadinBB*60 group and *Coriobacteriaceae* were significantly reduced (p < 0.0129 and p < 0.025, respectively). In the CTS group (Figure 6G), the enrichment of *Pasteurellaceae* was partially reversed (p < 0.0132), while significant differences were observed in *Cyanobiaceae* (p < 0.0412) compared to the Model group.

At the genus level (Figures 6H,I), *Rodentibacter* was highly enriched in the Model group compared to Control (p < 0.0013), whereas *Clostridia vadinBB60* group and *Collinsella* were significantly reduced (p < 0.0129 and p < 0.0139, respectively). CTS treatment significantly reduced *Rodentibacter* abundance (p < 0.0013) and promoted partial recovery of *Clostridia vadinBB60* group and *Collinsella* compared to the Model group. Significant differences in *Turicibacter* (p < 0.0158) and *Streptococcus* (p < 0.0411) were also noted.

All in all, these results reveal disease model-induced disruptions in gut microbiota composition, characterized by altered relative abundances of specific taxa. CTS treatment partially restored the microbial community composition, shifting it closer to Control levels.

### 3.6 CTS treatment can partially reverse the metabolic disturbances induced by tumor progression

**FIGURE 7:**
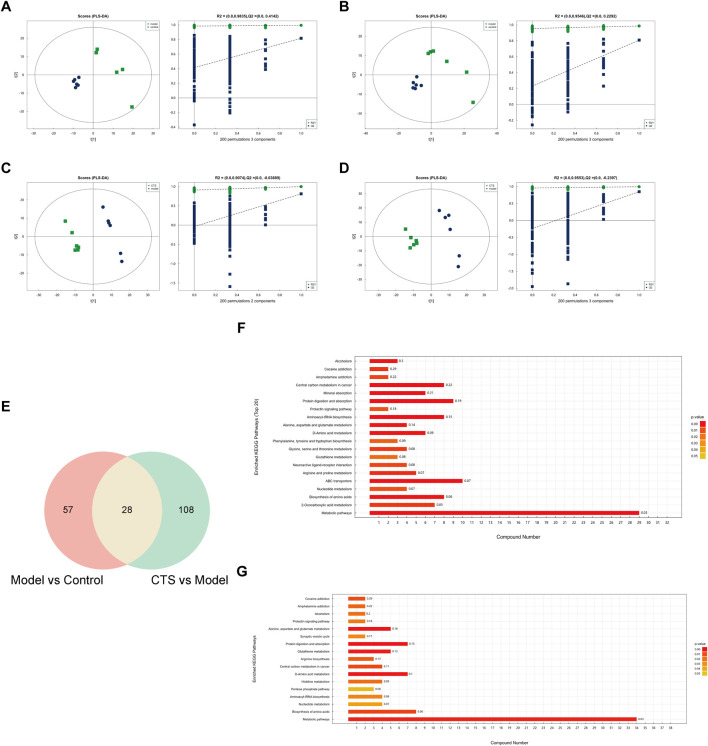
Metabolic alterations and pathway enrichment analysis of CTS treatment in CRC mice (n = 6). **(A–D)** PLS-DA score plots and permutation test results for the Model vs. Control **(A,B)** and CTS vs. Model **(C,D)** comparisons. The score plots show distinct separations between groups, while the permutation test confirms the reliability of the PLS-DA models. **(E)** Venn diagram showing the overlap of differential metabolites between the Model vs. Control and CTS vs. Model comparisons. **(F,G)** KEGG pathway enrichment analysis of differential metabolites in the Model vs. Control **(F)** and CTS vs. Model **(G)** groups.

To investigate the metabolic alterations associated with CTS treatment in CRC mice, untargeted metabolomics combined with multivariate statistical analysis was performed. PLS-DA score plots (Figures 7A–D) demonstrated distinct clustering between the Control, Model, and CTS groups, indicating significant differences in their metabolic profiles.

In the Model vs. Control comparison (Figures 7A,B), clear separation was observed, highlighting substantial metabolic reprogramming associated with CRC development. Similarly, in the CTS vs. Model comparison (Figures 7C,D), the CTS-treated group exhibited a distinct metabolic profile, reflecting the therapeutic impact of CTS on CRC-related metabolic disturbances. Permutation tests confirmed the robustness of the PLS-DA models, with high R^2^ values indicating goodness-of-fit and Q^2^ values supporting predictive reliability.

Differential metabolites were identified, and pathway enrichment analysis was performed to explore the metabolic changes associated with CTS treatment in CRC mice. The Venn diagram (Figure 7E) shows an overlap of metabolites from the Model vs. Control and CTS vs. Model comparisons, with 57 metabolites unique to CRC progression, 108 specifics to CTS treatment, and 28 shared metabolites potentially linked to both CRC progression and CTS-mediated effects.

Pathway enrichment analysis was conducted using the KEGG database to identify significantly altered metabolic pathways. In the Model vs. Control group (Figure 7F), enriched pathways included, central carbon metabolism in cancer, mineral absorption and protein digestion and absorption, indicating dysregulation of core energy metabolism and nutrient handling during CRC development.

Notably, in the CTS vs. Model group (Figure 7G), several cancer-relevant pathways were significantly enriched. These included glutathione metabolism, arginine biosynthesis, central carbon metabolism in cancer, and various amino acid metabolism pathways. This pattern suggests that CTS treatment counteracts CRC progression by modulating oxidative stress response, amino acid availability, and cancer cell energy metabolism.

A total of 969 metabolites were detected by LC-MS in combined positive and negative ion modes, of which 345 compounds were detected in positive ion mode and 1053 compounds were detected in negative ion mode, of which 29.185% were compounds of lipids and lipid-like molecules, 22.74% organic compounds and derivatives, 6.867% phenylpropanoids and polyketones, benzene compounds accounted for 6.81%.

**FIGURE 8:**
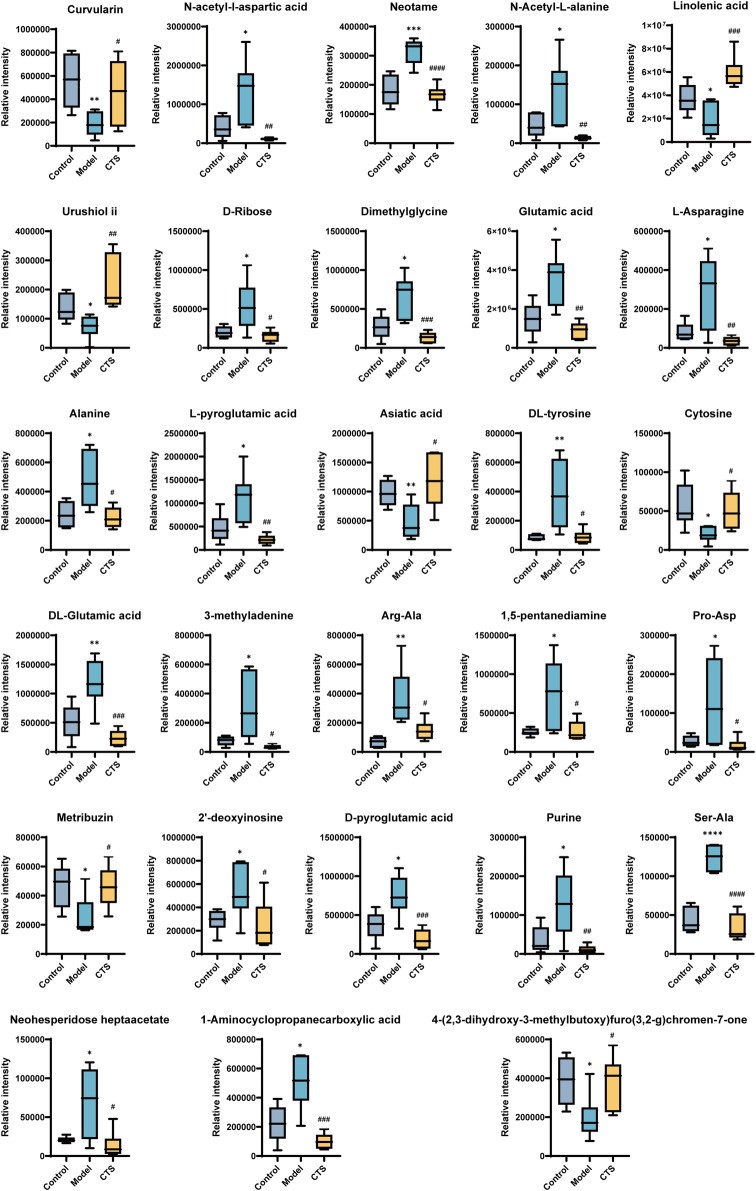
Metabolic profiling in CRC mice (n = 6).

The metabolic profiles of the Control, Model, and CTS groups were analyzed, revealing significant metabolic changes (Figure 8). In the Model group, compared to the Control group, several metabolites showed marked changes, reflecting the altered metabolic landscape associated with tumorigenesis.

In the Model group, metabolites such as Curvularin, Urushiol ii, Neotame, 3-methyladenine, 1,5-pentanediamine, Purine, and Metribuzin were significantly increased, suggesting enhanced polyamine and nucleotide metabolism, both of which are characteristic features of cancer progression. On the other hand, several metabolites involved in amino acid and nucleotide metabolism, such as N-acetyl-L-aspartic acid, N-acetyl-L-alanine, L-asparagine, Dimethylglycine, Cytosine, DL-glutamic acid, and Alanine, showed a significant decrease, indicating metabolic dysregulation in the Model group.

After CTS treatment, a partial reversal of the metabolic disturbances was observed. The levels of Glutamic acid, Dimethylglycine, N-acetyl-L-alanine, Alanine, and L-pyroglutamic acid were significantly increased in the CTS group compared to the Model group, suggesting that CTS may restore disrupted amino acid metabolism. Additionally, Neotame, DL-tyrosine, Asiatic acid, and L-pyroglutamic acid levels were significantly higher in the CTS group, indicating a potential normalization of certain metabolic pathways.

Moreover, CTS treatment had a significant impact on nucleotide metabolism. Purine, 2′-deoxyinosine, and D-pyroglutamic acid levels were significantly reduced in the CTS group compared to the Model group, suggesting that CTS could reverse the increased nucleotide metabolism observed in the Model group. The levels of Neohesperidoside heptaacetate and 1-Aminocyclopropanecarboxylic acid were significantly elevated in the CTS group, indicating the potential for CTS to regulate polyamine and nucleotide synthesis.

These metabolic changes suggest that CTS treatment can partially reverse the metabolic disturbances induced by tumor progression, particularly in amino acid, nucleotide, and polyamine metabolism, helping to restore metabolic balance.

## 4 Discussion

CTS, a lipophilic compound from *Salvia miltiorrhiza*, exhibits broad pharmacological activities, including anti-inflammatory and anticancer effects (Kitamura et al., 2010; Suzuki et al., 2011). Its potential as a therapeutic agent against CRC is supported by previous reports of Stat3 pathway inhibition and endoplasmic reticulum stress-induced (Fu et al., 2021; Li et al., 2015). Nonetheless, its comprehensive mechanism, particularly involving the tumor-immune-microbiome axis, warrants further elucidation.

Our study demonstrates that CTS significantly inhibits the proliferation of MC38 cells and the growth of mouse-derived CRC organoids, with effects evident at concentrations ≥5 µM. These *in vitro* findings were corroborated *in vivo*, where CTS treatment alleviated pathological features—including mucosal ulceration, inflammatory infiltration, and tumor formation—in AOM/DSS-induced mice. The collective ability of CTS to inhibit proliferation, induce apoptosis, and disrupt organoid formation in our models suggests a multi-faceted mechanism contributing to its anti-CRC activity.

Chronic inflammation, driven by pro-inflammatory cytokines such IL-6, TNF-α, and IL-1β, plays a pivotal role in CRC pathogenesis (Muthusami et al., 2021). Specifically, IL-6 contributes to tumor cell proliferation and the establishment of an immunosuppressive microenvironment (Heichler et al., 2020). TNF-α can activate the NF-κB pathway, which induces pro-inflammatory gene expression and promotes processes such as tumor cell invasion and angiogenesis (De Simone et al., 2015). Meanwhile, IL-1β is recognized for exacerbating colonic inflammation and supporting tumor microenvironment remodeling, mechanisms that are linked to the stimulation of the NLRP3 inflammasome (Zhan et al., 2024). The abnormal elevation of these cytokines is thought to not only sustains chronic inflammation but also accelerate CRC progression by promoting DNA damage, enriching cancer stem cells, and facilitating immune evasion.

In our AOM/DSS-induced model, we confirmed significant elevations in IL-6, IL-1β, and TNF-α levels. CTS treatment markedly reduced these cytokines, demonstrating its ability to alleviate intestinal inflammation and disrupt a key driver of CRC progression. This anti-inflammatory effect aligns with the documented properties of CTS.

The immune checkpoint CD279 (PD-1), upon engaging its ligands on tumor cells, transmits inhibitory signals that lead to T cell exhaustion and immune evasion (Liu et al., 2020). In our model, while overall T cell subpopulation frequencies were unchanged, CTS treatment significantly reduced the proportion of PD-1+ T cells. This reduction in PD-1+ T cells indicates an alleviation of T cell exhaustion, which could potentially contribute to improved anti-tumor immunity. Furthermore, we assessed the proliferation marker Ki-67, which was highly expressed in model mice but substantially suppressed after CTS treatment. Together, these data suggest that CTS combats CRC through integrated mechanisms involving immune modulation and direct suppression of tumor cell proliferation.

Alterations in the gut microbiota and microbiome are implicated in numerous diseases, including Crohn’s disease, autism, diabetes, cancer, and obesity (Cantón et al., 2024). Due to the specific anatomical location of CRC, the gut microbiota, as a crucial environmental factor, is closely related to the occurrence and progression of CRC. The combined action of *Escherichia coli* and *Bacteroides fragilis* can inhibit the expression of mucin, thereby weakening the protective function of the intestinal barrier and increasing the damage to intestinal epithelial cells caused by toxic substances (Wong and Yu, 2019).

Yachida et al. found that a decrease in the abundance of *Alistipes* and an increase in *Megasphaera* abundance were significantly associated with CRC progression (Yachida et al., 2019). The colonization of *Alistipes* in CRC mouse models can accelerate tumor progression, and it has been identified as a potential bacterial biomarker for clinical CRC diagnosis (Dai et al., 2018). Furthermore, the gut microbiota can promote cancer cell proliferation and metastasis by modulating inflammatory signaling pathways. For instance, *Bacteroides fragilis* has been shown to activate the NF-κB pathway, sustaining intestinal inflammation and thereby promoting the process of carcinogenesis (Chung et al., 2018).

Our study found that CTS altered the community structure of gut microbiota and regulated the relative abundance of certain bacterial taxa, including beneficial bacteria such as *Firmicutes*, *Lactobacillus*, and *Muribaculaceae*, as well as potentially harmful bacteria such as *Bacteroidota*, *Pasteurellaceae*, *Escherichia-Shigella*, and *Streptococcus*.

The therapeutic implication of this shift is underscored by the established roles of these bacteria in colorectal carcinogenesis. The ratio of *Firmicutes* to *Bacteroidota*, which was favorably altered by CTS, is a key indicator of gut ecosystem health and is often disrupted in inflammatory bowel disease and CRC (Stojanov et al., 2020). This is critical because beneficial members of the *Firmicutes* phylum, such as *Lactobacillus* and *Bifidobacterium*, are primary producers of short-chain fatty acids (SCFAs) like butyrate, which are instrumental in maintaining intestinal barrier integrity, resolving inflammation, and regulating immune responses that are typically compromised in CRC. The anticancer potential of *Lactobacillus* is well-documented, with mechanisms including the inhibition of cancer cell proliferation, induction of apoptosis, and immunomodulation. Supporting this, *Lactobacillus* supplementation has been shown to reduce pro-inflammatory cytokines and inhibit tumor formation in pre-clinical models (Chou et al., 2020), and oral administration of *Lactobacillus* casei can reduce tumor incidence in clinical settings (Ishikawa et al., 2005).

Conversely, the bacterial taxa suppressed by CTS is often implicated in driving carcinogenesis. Within the *Bacteroidota* phylum, virulent strains of *Bacteroides fragilis* can promote tumorigenesis by secreting toxins that damage the colonic epithelium and trigger pro-inflammatory responses (Kostic et al., 2012). Similarly, pathobionts like *Escherichia-Shigella* can induce DNA damage through the production of colibactin and compromise the intestinal barrier, while certain *Streptococcus* species can foster a procarcinogenic, hypoxic tumor microenvironment (Wong and Yu, 2019).

Our untargeted metabolomics analysis revealed that the anti-CRC effects of CTS are associated with the modulation of core metabolic pathways. We interpreted these findings with a focus on pathways that have established relevance to cancer biology. The Model group exhibited significant enrichment in central carbon metabolism in cancer and various amino acid metabolism pathways, underscoring the metabolic reprogramming that fuels tumor growth. Notably, CTS intervention was associated with a reversal of these changes. The modulation of glutathione metabolism is consistent with a restoration of redox balance, while the impact on central carbon metabolism suggests a potential disruption of energy supply in cancer cells. Furthermore, the shifts in amino acid metabolism are consistent with a model in which CTS may limit the availability of biosynthetic precursors essential for tumor proliferation.

Analysis of differential metabolites provided further mechanistic insights. The Model group showed elevated levels of metabolites associated with heightened nucleotide and polyamine metabolism, including Purine, Curvularin, and Neotame, which are characteristic of proliferative tumors. CTS treatment partially reversed this dysregulation, increasing levels of amino acids such as Glutamic acid, Dimethylglycine, and L-pyroglutamic acid, while reducing nucleotide metabolites including Purine and 2′-deoxyinosine. These metabolic shifts suggest that the anti-tumor effects of CTS may be mediated, in part, by the restoration of metabolic homeostasis in the tumor microenvironment. Collectively, the ability of CTS to coordinately modulate central carbon metabolism, redox balance, and nucleotide/amino acid availability suggests that its anti-tumor efficacy may involve a multi-faceted impact on cancer metabolism. Despite the compelling evidence presented herein, this study has limitations, including the use of a sample size that may limit the statistical power for some analyses. Nevertheless, the coordinated effects of CTS on tumor growth, immune exhaustion, gut microbiota, and host metabolism are consistent with a multi-targeted mechanism involving the tumor-immune-microbiome interface.

## 5 Conclusion

In conclusion, our study demonstrates that CTS exhibits significant anti-cancer effects in CRC by directly inhibiting cell proliferation and organoid formation. Furthermore, integrated multi-omics analysis reveals that CTS intervention is associated with a reversal of metabolic dysregulation, a reduction in inflammatory cytokines, and beneficial alterations in gut microbiota composition. These findings collectively highlight the potential of CTS as a therapeutic candidate for CRC and warrant further investigation. Future work should focus on elucidating the precise molecular mechanisms underlying these multi-target effects to fully unlock the therapeutic potential of CTS.

## Data Availability

The datasets presented in this study can be found in online repositories. The names of the repository/repositories and accession number(s) can be found in the article/supplementary material.
